# Stalk formation of *Brevundimonas* and how it compares to *Caulobacter crescentus*

**DOI:** 10.1371/journal.pone.0184063

**Published:** 2017-09-08

**Authors:** Patrick D. Curtis

**Affiliations:** Department of Biology, University of Mississippi, University, MS, United States of America; Centre National de la Recherche Scientifique, Aix-Marseille Université, FRANCE

## Abstract

The *Caulobacter crescentus* cell extension known as a stalk represents an unusual bacterial morphology. *C*. *crescentus* produces stalks under multiple nutrient conditions, but the length of the stalk is increased in response to phosphate starvation. However, the exact function of the stalk is not known, nor is it known how much stalk biogenesis or function is conserved with other stalked bacteria. Work presented here shows that many organisms in the *Caulobacter* genus and the next closest genus (*Brevundimonas*) generally do not synthesize stalks in the relatively-rich PYE growth medium, suggesting that the synthesis of a stalk under nutrient-rich conditions by *C*. *crescentus* may be the exception instead of the norm among its phylogenetic group. *Brevundimonas subvibrioides* can be induced to synthesize stalks by genetically mimicking phosphate starvation conditions, indicating stalk synthesis in this organism may be performed on an as-need basis. This mutation, however, does not appear to increase the incidence of holdfast synthesis. While *B*. *subvibrioides* stalks appear to be synthesized with the same polarity with respect to holdfast as *C*. *crescentus* stalks, evidence is presented that suggests *B*. *subvibrioides* may disassemble stalks when they are no longer needed. Many homologs of *C*. *crescentus* genes encoding stalk-associated proteins are absent in the *B*. *subvibrioides* genome, and *B*. *subvibrioides* PstA-GFP as well as *C*. *crescentus* StpX-GFP are able to enter the *B*. *subvibrioides* stalk compartment, calling into question the level of compartmentalization of the *B*. *subvibrioides* stalk. In summary, this work begins to address how much the *C*. *crescentus* model for this unusual morphological adaptation can be extended to related organisms.

## Introduction

The prosthecate, or stalked, bacteria are a group of bacteria that create thin extensions of the cell wall from the main cell body. Many stalked bacteria are found in the *Alphaproteobacteria* clade, including *Caulobacter*, *Brevundimonas*, *Asticcacaulis* and *Hyphomonas* genera. Stalks and stalked bacteria have been of research interest for a number of reasons. Stalks are generally restricted to specific spatial regions of the cell (usually polar) and are therefore useful markers for studying subcellular localization programs in bacteria. As an unusual morphological event, stalks also are a target of study for trying to understand the fundamental biology of cell shape; i.e. how and why bacterial cells alter their shape. Therefore, understanding the regulation and synthesis of stalks can inform multiple areas of research.

The most well-characterized model for stalk synthesis is in the developmental organism *Caulobacter crescentus*. An occupant of freshwater oligotrophic environments, *C*. *crescentus* has a dimorphic life cycle (reviewed in [1]). The *C*. *crescentus* life cycle begins as a “swarmer cell”; a replication incompetent cell motile via a single polar flagellum. In order to begin cellular replication, the swarmer cell must differentiate into a “stalked cell”, which is typically characterized by ejecting the polar flagellum and synthesizing a stalk at the same pole, along with changes to intracellular signaling systems. In some conditions, the stalk is tipped with an adhesive polysaccharide called a holdfast. As the stalked cell grows and prepares for division it is referred to as a “predivisional cell”. In the predivisional cell stage a flagellum is synthesized on the pole opposite the stalk; this results in the two daughter cells inheriting different polar structures after division. The stalked daughter cell is able to re-enter the replication cycle, while the flagella-inheriting daughter cell is initially replication incompetent and, as stated, must differentiate into a stalked cell to enter S-phase. This complex life cycle is coordinated by a number of intricate intracellular signaling networks that spatially and temporally control both morphological differentiation as well as replication competency.

*C*. *crescentus* has two different programs of stalk synthesis. One program, here referred to as the developmental program, occurs naturally during the progression of the life cycle. However, in response to phosphate starvation *C*. *crescentus* synthesizes extremely long stalks, as much as ten times the length of the cell body [2–4]. This phosphate response appears to be a separate synthesis program as *C*. *crescentus* mutants that do not produce stalks as part of the life cycle in nutrient rich conditions will synthesize stalks when starved for phosphate [5–7]. In fact, in the related organism *Asticcacaulis excentricus* these two programs appear to be morphologically distinct, where developmental stalks are sub-polarly localized but stalks produced during phosphate starvation are polarly localized [8]. Therefore, this second program will be here referred to as the phosphate-starvation program. The synthesis of stalks in response to phosphate starvation is known to employ the Pho regulon [2]. Mutants of the *pstS* gene in *C*. *crescentus* mimic phosphate starvation and produce long stalks even in phosphate replete conditions, while mutants of the response regulator *phoB* block the Pho regulon and make short stalks even under phosphate starvation conditions.

Genomic analysis has revealed that the intracellular signaling networks that coordinate *C*. *crescentus* development are conserved in whole or part throughout the alphaproteobacterial clade, suggesting that the work characterizing the *C*. *crescentus* model may be extrapolated to a large number of organisms [9]. However, recently it has been shown that there are substantial differences in the essentiality and operation of a number of developmental regulators between *C*. *crescentus* and *Brevundimonas subvibrioides* [10]. *Brevundimonas* is the next closest genus to *Caulobacter* and the organisms are found in the same oligotrophic environments, therefore it was surprising to find fundamental differences in functionality between the organisms. To further compare the biology of these two organisms, stalk synthesis was investigated in other *Caulobacter* and *Brevundimonas* species, and in *B*. *subvibrioides* in greater detail. Results presented here show that stalk synthesis under nutrient-rich conditions, as exemplified by *C*. *crescentus*, appears rare among these groups, and that *B*. *subvibrioides* appears to synthesize stalks more on an as-need basis. While many known stalk-associated proteins are not conserved between the species, *B*. *subvibrioides* may have a conserved mechanism for permitting certain proteins to enter the stalk compartment. This work begins to address the question of how much the *C*. *crescentus* stalk model is applicable to related organisms.

## Materials and methods

### Bacterial strains and growth conditions

A list of strains is included in S1 Table. *Escherichia coli* was cultured on LB medium (10 g/L tryptone, 5 g/L yeast extract, 10 g/L NaCl) at 37^°^C. Where necessary, media was supplemented with kanamycin at 50 μg/ml. *Caulobacter* and *Brevundimonas* strains were cultured in PYE medium [11] or 2X PYE at 30^°^C, which the exception of *Caulobacter* sp. K31 which was cultured in PYE supplemented with 10 μg/ml riboflavin. Where necessary, media was supplemented with kanamycin at 10 μg/ml and/or tetracycline at 4 μg/ml. PYE plates containing 3% sucrose were used for counter-selection. *C*. *crescentus* was also cultured in HIGG medium with a final phosphate concentration of 30 μM [12]. Where needed, vanillate (pH 7.5) was added to the media at a final concentration of 500 μM. This value was based on induction levels investigated in *C*. *crescentus* [13].

To perform growth curves, overnight wild-type and Δ*pstS* strains were inoculated into 25 ml PYE in triplicate such that all cultures had an initial to OD_600_ = 0.009, as measured on a Nanodrop 2000 spectrophotometer (the Nanodrop 2000 uses a 1 mm light path so all optical density measurements are an order of magnitude smaller than a standard 1 cm light path spectrophotometer). Cultures were shaken 200 rpm at 30^°^C for 24 hours, and 100 μl aliquots were removed every hour for optical density measurement.

### Microscopy conditions

In order to determine the percentage of stalk incidence for different strains under nutrient-rich conditions, *Caulobacter* and *Brevundimonas* strains were grown to mid-exponential phase (OD_600_ = 0.04–0.06) in PYE media, and 0.5 μl aliquots were spotted onto pads composed of 1.0% agarose in water. Cells were viewed by phase contrast microscopy and 10 images of each slide were obtained. All cells were scored for the presence or absence of stalks. As flagella and pili are typically not visible under phase contrast conditions, any observable extension from a cell pole was considered a stalk (with no required minimum size), and the percent of stalk incidence was calculated by dividing the number of stalked cells by the total cell count for that slide. Multiple planes of focus were analyzed to determine the plane that best visualized stalks of a given sample. Each strain was analyzed by this procedure three times, and the average and standard deviation of stalk incidence was calculated.

Holdfast staining was based on the protocol of [14]. Alexafluor 488 (GFP imaging conditions) or Alexafluor 594 (RFP imaging conditions) conjugated to Wheat Germ Agglutinin (WGA) were commercially purchased (Molecular Probes) and resuspended in phosphate-buffered saline (pH 7.4) to a concentration of 5 mg/ml. Mid-exponential phase cultures (200 μl) were mixed with 2 μl WGA and incubated for 20 min at room temperature. One milliliter of water was added to the cell suspension, then the cells were centrifuged 15,000 x g for 1 min at room temperature. The supernatant was removed and the cells were resuspended in 30 μl water. Cells (0.5 μl) were spotted onto pads of 1.0% agarose in water. Cells were analyzed by phase contrast and epifluorescence microscopy at appropriate wavelengths. For holdfast incidence quantification, each strain was cultured and labeled three times, and 10 images were obtained per labeled sample. Cells were scored on whether they had an observable holdfast, stalk, both, or neither. Stalks were scored on phase contrast images first as holdfast fluorescence can sometimes obscure short stalks. Averages and standard deviations were calculated based on the triplicate samples.

For *B*. *subvibrioides* time lapse analysis, strains were grown to mid-exponential phase in PYE media and 0.5 μl were spotted on pads composed of 1.0% agarose in 2X PYE. The higher nutrient concentration of 2X PYE was used to compensate for the lower growth temperature used during time-lapse microscopy. *B*. *subvibrioides* has been previously shown to grow in 2X PYE [10], and the doubling time reduces from 6.5 hrs in PYE to 4.5 hrs in 2X PYE at 30^°^C, showing a growth stimulatory effect (S. Adhikari and P.D. Curtis, unpublished). Images of cells under phase contrast microscopy were recorded every 30 minutes for 8 hours. For *C*. *crescentus* time lapse analysis, cells were grown to mid-exponential phase in HIGG low-phosphate media or PYE, and then spotted onto pads composed of 1.0% agarose in 2X PYE. Images of cells under phase contrast microscopy were recorded every 30 minutes for 8 hours.

GFP-labeled strains were grown to mid-exponential phase, 0.5 μl were spotted on 1.0% agarose/water pads and analyzed by phase contrast and epifluorescence microscopy at appropriate wavelengths. For *stpX-gfp* expression experiments, either the control vector pRVGFPC-2 or pPDC24 (see below) were transformed into wild-type or Δ*pstS B*. *subvibrioides*. Strains were grown to mid-exponential phase in the absence or presence of 500 μm vanillate (pH 7.5). Cells (0.5 μl) were spotted on 1.0% agarose/water pads and analyzed by phase contrast and epifluorescence microscopy at appropriate wavelengths.

All phase contrast or epifluorescence images were taken using the same magnification and cropped to the same size without resizing, therefore sizes are comparable across images. All GFP-labeled or WGA-stained strains were imaged with the same exposure conditions and fluorescence intensity was scaled the same.

For Transmission Electron Microscopy, each culture was grown to mid-exponential phase and DMSO was added to a final concentration of 10% prior to freezing at -80^°^C. Samples were then shipped to the Center for Advanced Microscopy (Michigan State University) for imaging. Samples were prepared by aliquoting a 5 μl drop of sample on to a 200 mesh carbon grid followed by staining with 1% uranyl acetate. Images were taken at 5,000X magnification. Cells were scored for the presence of stalks (with any observable polar extension considered a stalk).

### Mutant construction

To create a deletion of *pstS* in *B*. *subvibrioides*, ~500 bp fragments upstream and downstream of the *pstS* gene were PCR amplified using primers pstForwardUp (GGGAAGCTTCTAGCCGTCTTCGCGGCGG), pstReverseUp (AGGGGATCCGGCGGGCATCAGCACAGTC), pstForwardDown (GCGGGATCCGAAAAATAGGGTTCAGGCCGCC), and pstReverseDown (GATGAATTCCAGCCTGGGGGGCAAGGACTGG). The fragments were cloned into pNPTS138 (M.R. Alley, unpublished), creating plasmid pPDC12, which can be used to create an in-frame deletion of *pstS* leaving only 7 codons. This plasmid was transformed into *B*. *subvibrioides* by electroporation [10], followed by kanamycin selection. Kanamycin resistant colonies were inoculated into PYE media and grown in the absence of selection, followed by plating on PYE sucrose plates to select for double recombinants; pNPTS138 has a *sacB* counter-selection marker. Resultant strains were screened for kanamycin sensitivity, and sensitive strains were analyzed by PCR to screen for *pstS* deletion.

To create a deletion of *phoB* in *B*. *subvibrioides*, ~500 bp fragments upstream and downstream of the *phoB* gene were PCR amplified using primers phoBForwardUp (ATCAAGCTTATCGCCCTGCGCCAGCCGGTC), phoBReverseUp (CACGGATCCATAGGGCTGCATGTCGGTCTC), phoBForwardDown (CTGGGATCCTGACGTCAGTCCGTCGCTTG), and phoBReverseDown (GTAGAATTCGCAGCCTTCGCCCGGATCGTC). The fragments were cloned into pNPTS138, creating plasmid pPDC13. Transformation, selection and counter-selection were performed as described above. Kanamycin sensitive strains were screened for *phoB* deletion by PCR. To express *phoB* from an ectopic inducible plasmid, the *B*. *subvibrioides phoB* gene, including stop codon, was PCR amplified using primers PhoBxylF (CTTCATATGCAGCCCTATATCCTGGTG) and PhoBvanR (GTTGCTAGCTCAGCCGTCCAGATCCAGGG), and cloned into plasmid pRVMCS-5 [13], creating plasmid pPDC26. This resulting plasmid was transformed into *B*. *subvibrioides* where the *phoB* deletion construct was already integrated into the chromosome, and transformants were selected for on PYE growth media containing kanamycin, tetracycline, and vanillate. Successful transformants were cultured in media containing tetracycline and vanillate, followed by counterselection on media containing tetracycline, vanillate, and sucrose. Double-recombinants were then screened for chromosomal *phoB* deletion.

To create a GFP fusion to *B*. *subvibrioides* PstA, the full *pstA* gene without the stop codon was PCR amplified using primers PstAGFPvanF (GAACATATGATGACTGAGGCGACCCCCGAC) and PstAGFPvanR (CTTCTTAAGCTCCACCGGCGTTCGAACCGC). This fragment was then cloned into pRVGFPC-2 [13], creating plasmid pPDC25. The resulting plasmid was transformed into WT and Δ*pstS B*. *subvibrioides* by electroporation, followed by kanamycin selection. This plasmid is a replicating plasmid where the *pstA* gene expression is under control of the vanillate promoter and the protein produced is C-terminally GFP-tagged. Vanillate induction of the control vector (pRVGFPC-2) results in production of soluble GFP.

To express the *C*. *crescentus stpX* gene in *B*. *subvibrioides*, the full *stpX* gene without the stop codon was PCR amplified using primers stpXxylF (AAACATATGTTTGGACGTAATATACGCTTG) and stpXvanR (CTTCTTAAGCTGTGGTGAGCGTGCTCAAGTTC). This fragment was then cloned into pRVGFPC-2 [13], creating plasmid pPDC24. The resulting plasmid was transformed into WT and Δ*pstS B*. *subvibrioides* by electroporation, followed by kanamycin selection. This plasmid is a replicating plasmid where the *stpX* gene expression is under control of the vanillate promoter and the protein produced is C-terminally GFP-tagged.

### Quantification of stalk fluorescence

Three independent cultures of Δ*pstS* strains expressing either GFP, PstA-GFP, or StpX-GFP were grown to mid-exponential phase in the presence of vanillate. Samples of each culture were mounted on 1% agarose pads as described above. Images were captured of at least 50 cells displaying both a visible stalk as well as cell body fluorescence from each culture; i.e. at least 150 cells total were used per strain. Grayscale 16-bit images were taken using identical conditions and were not manipulated. Quantification of fluorescence was performed using ImageJ software [15], based on the protocol of [16]. A square Region of Interest was defined that contained only the stalk of a given cell, and the Integrated Density was measured. The Region of Interest was then moved to an area of the image immediately adjacent to the cell and the Mean Grayscale and Area were measured. The Total Corrected Fluorescence was calculated by: Integrated Density–(Area x Mean Grayscale). This provides the total fluorescence of the area subtracting out background fluorescence. The same steps were used with the Region of Interest capturing the cell body without the stalk. Additionally, measurements of two background areas were taken from over 150 different images and used to calculate Total Corrected Fluorescence values as to provide the variance of background fluorescence. Two-tailed Students T-test assuming unequal variance was used to compare the stalk fluorescence of different GFP constructs to background fluorescence to determine if the fluorescent signal in stalks of a given strain were statistically significant above background.

## Results

### Stalk biogenesis appears rare for many species under nutrient-rich conditions

**Fig 1:**
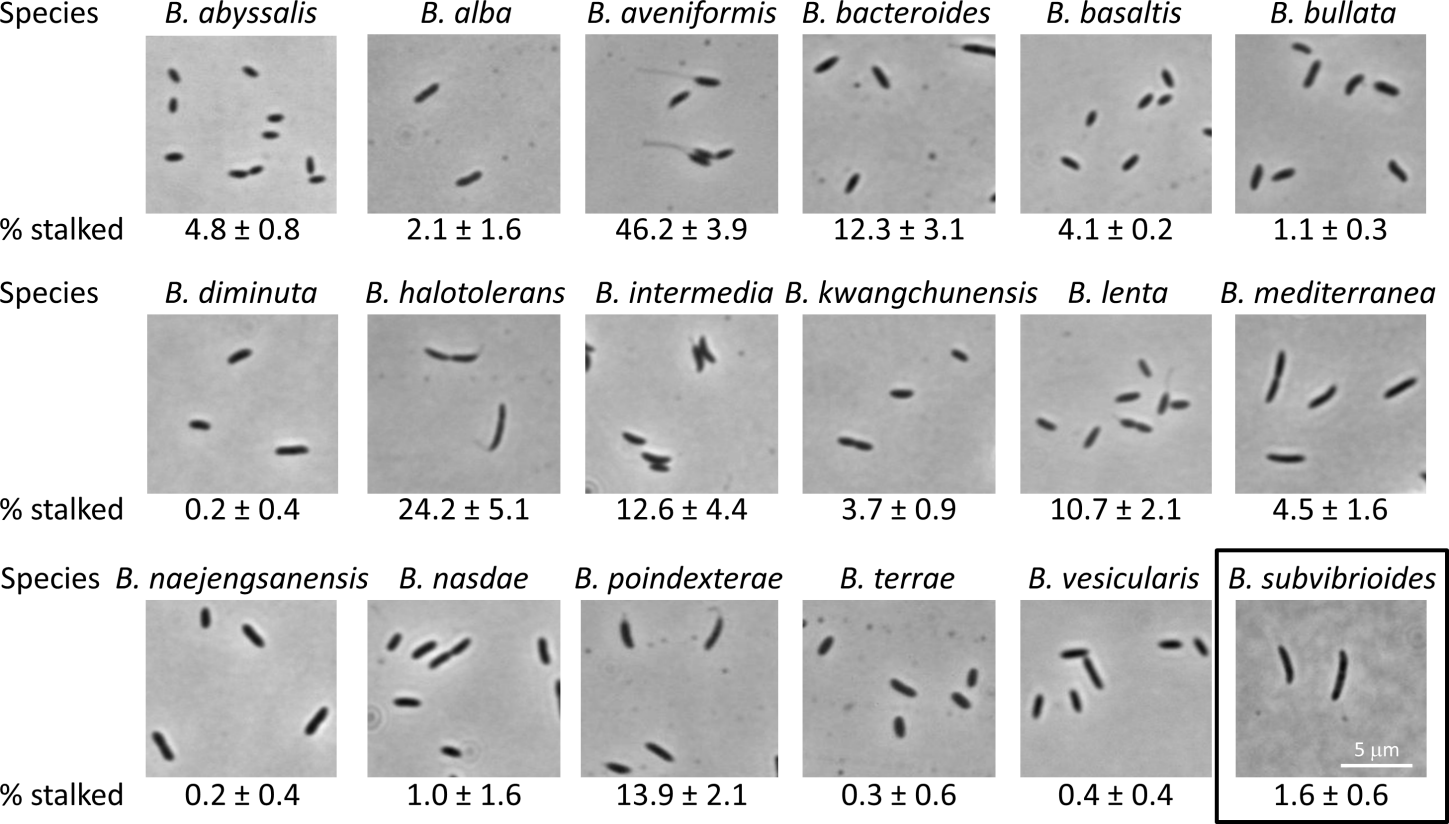
Visible stalk formation is rare among *Brevundimonas* species. *Brevundimonas* species were cultured in PYE medium to mid-exponential growth phase, 10 images were taken of cells and the percentage of total cells bearing a visible stalk was calculated. This process was repeated three times and used to determine the average and standard deviation (% stalked). Between 454 and 3174 cells were counted for each species, with most species having over 1000 cells counted.

One of the most useful aspects of *C*. *crescentus* physiology is the production of a unipolar stalk. This appendage provides rapid identification of the old pole after cell division, and is an orientation marker for asymmetrically distributed proteins in the cell. Previous analysis of *B*. *subvibrioides* indicated that most cells do not produce stalks in PYE growth medium, which is a relatively rich medium compared to the oligotrophic environments *C*. *crescentus* and *B*. *subvibrioides* typically inhabit. PYE presumably has non-limiting levels of phosphate [2], and will hereafter be referred to as nutrient-rich conditions. To quantitatively determine the incidence of stalk biogenesis in the *Brevundimonas* genus, 18 different *Brevundimonas* species were cultured in PYE medium and analyzed by phase contrast microscopy for stalk biogenesis (Fig 1); hereafter, “visible stalks” will refer to stalks visible by phase contrast microscopy. The number of visible-stalk-bearing cells as a percentage of the total population was calculated for each species.

As expected, *B*. *subvibrioides* had a very low proportion of visible-stalk-bearing cells with only 1.6 +/- 0.6% of cells having a visible stalk. In fact, only 6 *Brevundimonas* species had above 10% cells with visible stalks, and only 2 species had above 20%. *Brevundimonas halotolerans* had 24.2 +/- 5.1% of cells with visible stalks, and *Brevundimonas aveniformis* had the highest proportion with 46.2 +/- 3.9% of cells with visible stalks. Interestingly, the stalks of *B*. *aveniformis* were extremely long, reminiscent of the stalks seen in phosphate-starved *C*. *crescentus* cultures (see below). These results suggest that under these growth conditions visible stalk formation occurs rarely in many *Brevundimonas* species. It is possible, perhaps even probable, that visible stalk formation could be increased for many of these species by culturing them under different growth conditions.

**Fig 2:**
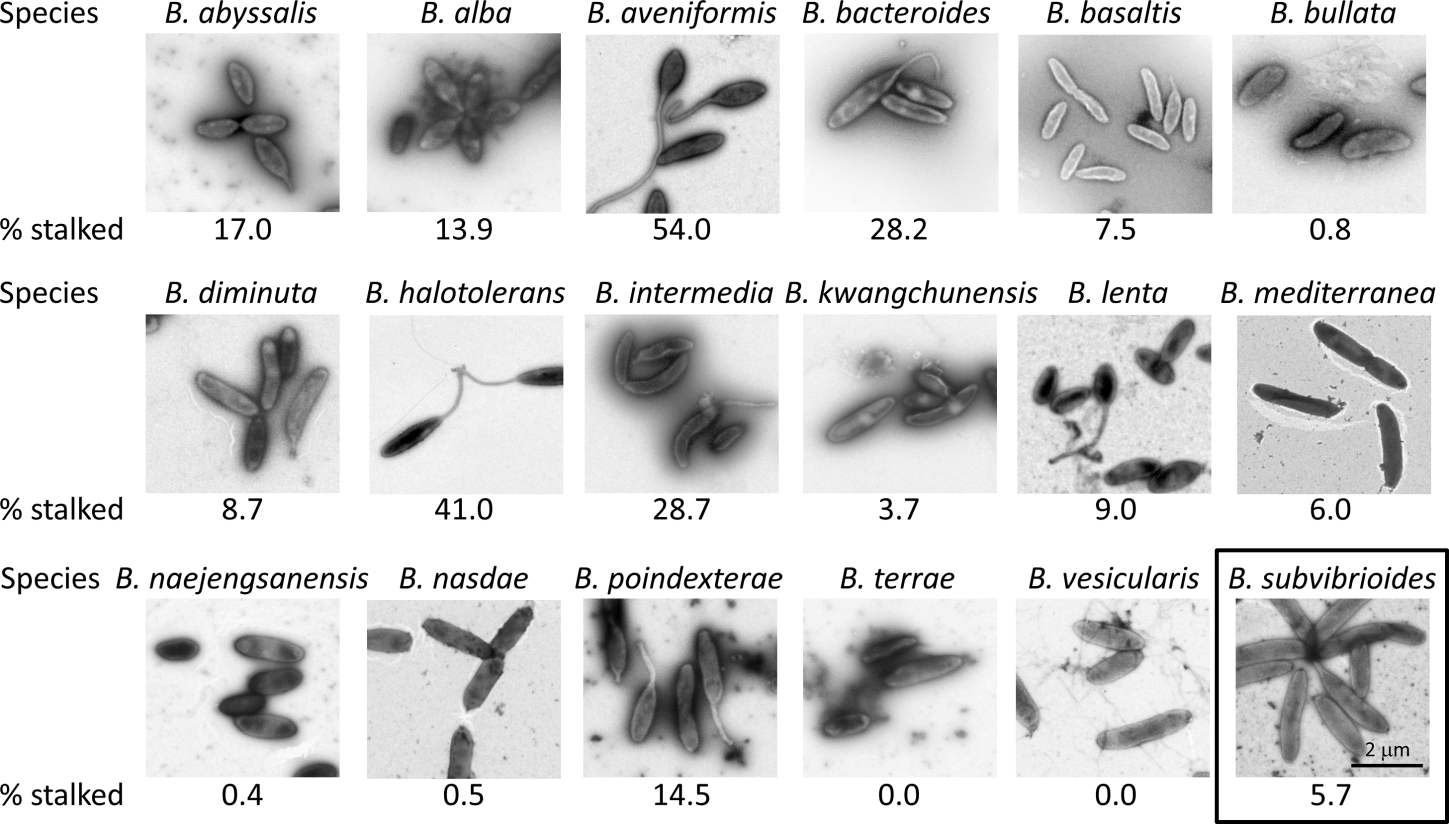
Transmission electron microscopy of *Brevundimonas* species. *Brevundimonas* species were cultured in PYE medium to mid-exponential growth phase and imaged by TEM. Cells were counted to determine the percentage of cells displaying polar extensions (% stalked). Between 92 and 248 cells were counted for each species. With some exceptions, percentages of stalked cells as determined by TEM were close to the number derived by phase contrast microscopy, supporting the results that these organisms produce stalks relatively rarely under nutrient rich conditions.

As small stalks can be difficult to visualize by phase contrast microscopy, transmission electron microscopy (TEM) was carried out on all *Brevundimonas* species and the percentage of cells bearing a stalk for each species was determined (Fig 2). There were some differences in results as *B*. *abyssalis*, *B*. *alba*, *B*. *aveniformis*, *B*. *bacteroides*, *B*. *halotolerans*, and *B*. *intermedia* had notably more stalked cells by percentage determined by TEM than by phase contrast microscopy. However, all the remaining species had percentages close to the phase contrast numbers, with most being within error of the percentages determined by phase contrast. More importantly, the same general trend was observed. By phase contrast analysis 12 of the 18 species had less than 10% of cells display visible stalks, and 16 of the 18 species had less than 20% of cells with visible stalks. By TEM analysis 11 of the 18 species had less than 10% of cells display stalks, and 14 of the 18 species had less than 20% of cells display stalks. Therefore, the TEM data supports the conclusion drawn from the phase contrast data that stalk formation is relatively rare among these *Brevundimonas* species grown in PYE medium. The TEM data also suggests that the phase contrast data is generally an accurate representation of stalk formation for most *Brevundimonas* species, including *B*. *subvibrioides*, which is important for much of the work presented here.

**Fig 3:**
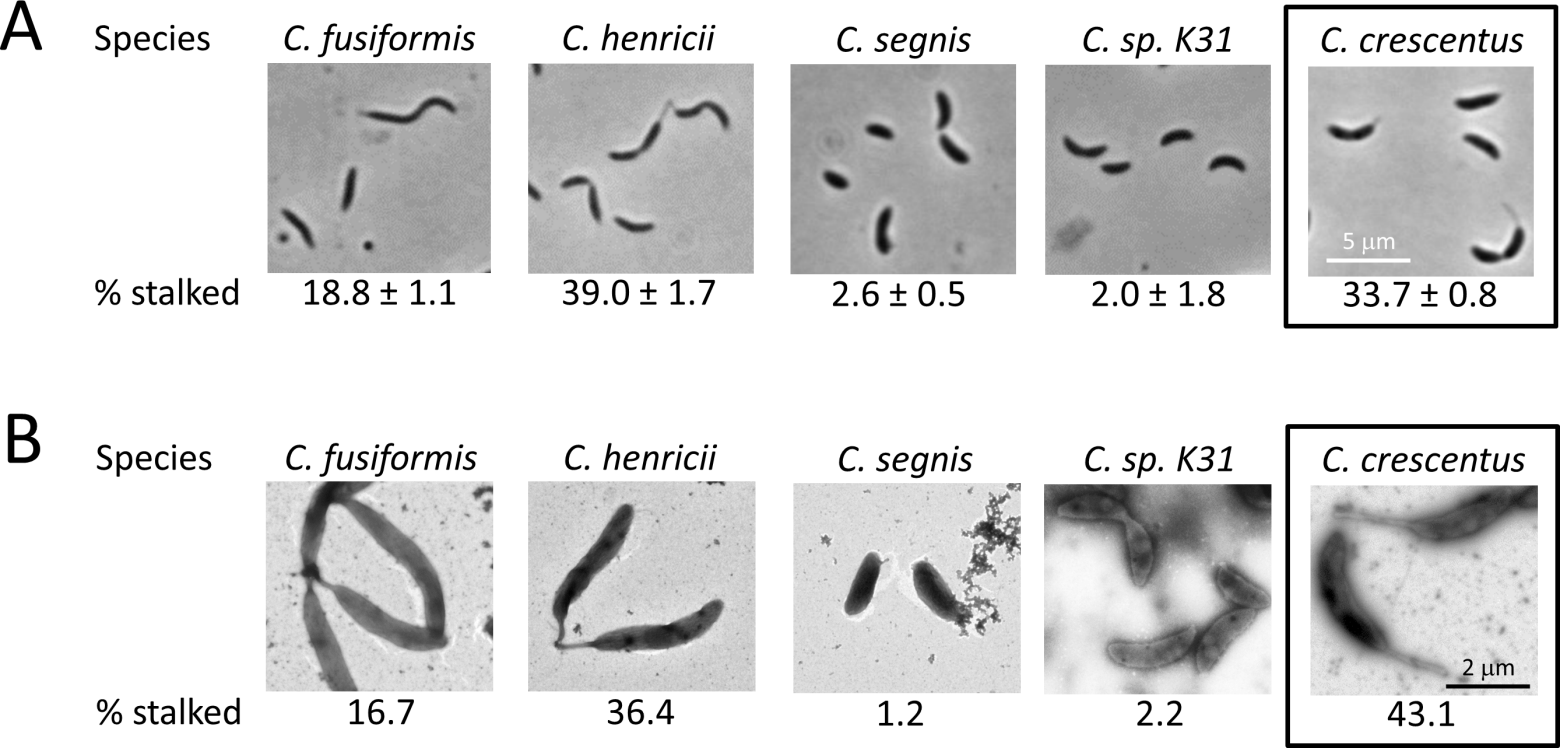
Stalk formation is also variable in some *Caulobacter* species. *Caulobacter* species were cultured in PYE medium to mid-exponential growth phase. A) 10 images were taken of cells and the percentage of total cells bearing a visible stalk was calculated. This process was repeated three times and used to determine the average and standard deviation (% stalked). Between 381 and 1138 cells were counted for each species. B) TEM imaging was performed on each strain and the percentage of cells displaying a polar extension was enumerated (% stalked). Between 83 and 151 cells were counted for each species.

When cultured under the same conditions, the wild-type *C*. *crescentus* NA1000 strain showed 33.7 +/- 0.8% of cells with a visible stalk by phase contrast (Fig 3A), which stands in contrast to the majority of *Brevundimonas* species analyzed. It may be that an increased propensity for stalk synthesis under nutrient-rich conditions is a trait common to the *Caulobacter* genus and could represent a distinction between the *Caulobacter* and *Brevundimonas* genera. To begin addressing this possibility, four other *Caulobacter* species were analyzed for the incidence of visible stalk synthesis under nutrient-rich conditions (Fig 3A). While *C*. *fusiformis* and *C*. *henricii* had larger proportions of their cell population demonstrate visible stalks, both *C*. *segnis* and *C*. *sp*. *K31* had below 3% of cells with visible stalks. Analysis by TEM resulted in percentages virtually identical to the phase contrast results for these four species (Fig 3B), and demonstrated a slightly higher percentage of stalked cells for *C*. *crescentus*. These results suggest that stalk formation under nutrient-rich conditions could be variable within the *Caulobacter* genus as well as the *Brevundimonas* genus. If this is true, then the propensity for stalk formation under nutrient-rich conditions of *C*. *crescentus* could be more the exception than the rule.

### Deletion of *pstS* causes a growth defect and increases visible stalk incidence but not holdfast incidence in *B*. *subvibrioides*

As PYE appears to have non-limiting levels of phosphate, the propensity for stalk formation of the species cultured in that medium is likely tied to the developmental program of stalk synthesis. However, this medium does not address the phosphate starvation program. Will phosphate starvation increase the incidence of visible stalks for organisms that have low incidence when cultured under nutrient-rich conditions, such as *B*. *subvibrioides*? There are two different mechanisms by which the phosphate starvation program may be analyzed in *C*. *crescentus*. The first is to culture the organism in phosphate-limited HIGG minimal medium [3, 4]. Phosphate starvation causes *C*. *crescentus* to synthesize extremely long stalks that can be many times the length of the cell body. Unfortunately, this method proved fruitless for *B*. *subvibrioides* as it has a yet-undiscovered auxotrophy that prevents growth in minimal media. HIGG growth media was supplemented with different nutrients (singly and in combination) commonly needed by natural auxotrophs, including biotin, nicotinic acid, thiamine, and riboflavin. Additionally, the genome was analyzed for potential amino acid auxotrophies leading to the testing of several different amino acids as well. No growth was observed in any trial (data not shown).

The other mechanism to induce stalk synthesis in *C*. *crescentus* is to disrupt the *pstS* gene [2]. PstS is the phosphate-chelating periplasmic protein that works with the high-affinity PstCAB transporter but it also participates in the control of the Pho regulon. Under phosphate-replete conditions, PstS spends more time bound to the transporter and subsequently leads to repression of the Pho regulon, while under phosphate starvation PstS spends more time away from the transporter and the Pho regulon is induced. Disruption of *pstS* mimics the phosphate starvation condition and leads to induction of the Pho regulon, which in *C*. *crescentus* leads to the formation of extremely long stalks. Conversely, disruption of the *phoB* response regulator disrupts Pho signaling, leading to constitutive inactivation. In *C*. *crescentus*, *phoB* disruption leads to short stalks even under phosphate starvation [2].

Previous TnSeq analysis of *B*. *subvibrioides* indicated that components of the Pst system as well as the PhoR/B two-component system are very important to the physiology of the organism [10]. The TnSeq study predicted that PhoB was essential. To test that hypothesis, a *phoB* deletion construct was created and moved into *B*. *subvibrioides*, followed by selection and then counter-selection to obtain double recombinants. If *phoB* was non-essential, double recombination should result in approximately half of the strains reverting to wild-type, while the other half should result in deletion. Over 70 different double recombinants (generated from multiple different outgrowth lineages from the parent strain and with extended counter-selection incubation times) all reverted to wild-type (data not shown). However, when a copy of *phoB* was placed on a replicating plasmid and expression induced using vanillate, the chromosomal *phoB* was easily deleted (data not shown). This result is consistent with *phoB* being essential to *B*. *subvibrioides*. This is in contrast to *C*. *crescentus* where *phoB* is non-essential.

**Fig 4:**
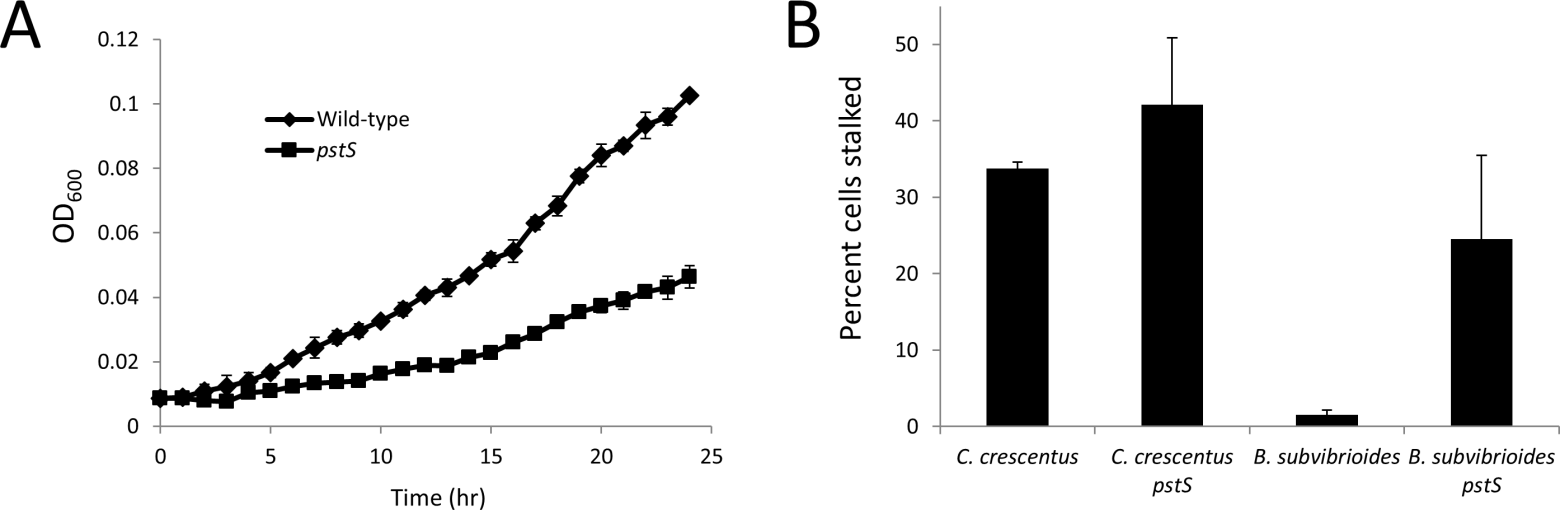
Deletion of *pstS* in *B*. *subvibrioides* leads to a growth defect and increased visible stalk biogenesis. A) Growth curve of wild-type and Δ*pstS B*. *subvibrioides* strains in PYE media. Wild-type had a doubling time of 6.5 hrs while Δ*pstS* had a doubling time of 8.6 hrs. B) Incidence of stalk formation as a percentage of total cell population in wild-type and Δ*pstS* disruption strains for *C*. *crescentus* and *B*. *subvibrioides*. Disruption of *pstS* increased visible stalk incidence from 33.7 +/- 0.8% to 42.1 +/- 8.8% in *C*. *crescentus*, and from 1.6 +/- 0.6% to 24.5 +/- 11.0% in *B*. *subvibrioides*.

In the *B*. *subvibrioides* Tnseq study *pstS* was characterized as “unresolved”, suggesting that it is either essential or its disruption leads to a growth defect [10]. A *pstS* deletion construct was made and introduced into *B*. *subvibrioides*, followed by selection and counter-selection. After counter-selection, many colonies arose on plates in a time frame consistent with wild-type growth, however all these strains were found to be wild-type revertants. Yet after prolonged incubation small colonies arose several days after the wild-type revertants. Testing these colonies revealed them to be *pstS* deletions and also suggested this deletion results in a growth defect. To determine the effect of *pstS* deletion on growth, growth curves were performed using wild-type and Δ*pstS B*. *subvibrioides* strains in PYE media (Fig 4A). While wild-type had a doubling time of 6.5 hrs (consistent with previous results [10]), the Δ*pstS* mutant had a doubling time of 8.6 hrs under the same conditions, demonstrating that deletion of *pstS* had a significant impact on the growth of the organism. This result is notable because a recent study suggested that TnSeq analysis does not produce the resolution needed to predict more subtle fitness changes caused gene disruption [17], yet the *B*. *subvibrioides* TnSeq study suggested that genes characterized as “unresolved” likely caused growth defects when disrupted [10], and thus accurately predicting the growth defect of the *B*. *subvibrioides* Δ*pstS* strain.

To determine the effect of *pstS* disruption on stalk incidence, the percentage of visible stalked cells was quantified for wild-type and *pstS* disruption strains in both *C*. *crescentus* and *B*. *subvibrioides*. In *C*. *crescentus*, *pstS* disruption led to a modest increase in visible stalk incidence, increasing the percentage of visibly stalked cells from 33.7 +/- 0.8% in wild-type to 42.1 +/- 8.8% in *pstS*::miniTn5 (Fig 4B). TEM analysis recapitulated these results with 43.1% stalked cells in wild-type and 57.5% stalked cells in the mutant. However, in *B*. *subvibrioides* the increase was more dramatic, going from 1.6 +/- 0.6% visibly stalked cells in wild-type to 24.5 +/- 11.0% in Δ*pstS*. Again TEM analysis recapitulated the results with 5.7% stalked cells in wild-type and 23.9% stalked cells in the mutant. Therefore, it appears phosphate starvation does increase visible stalk incidence in *B*. *subvibrioides*, and suggests a model where stalk formation in *B*. *subvibrioides* is performed on a more as-need basis than in *C*. *crescentus*. It is unknown if other *Brevundimonas* species that do not appear to synthesize stalks a high rate under nutrient rich conditions also behave in this fashion.

**Fig 5:**
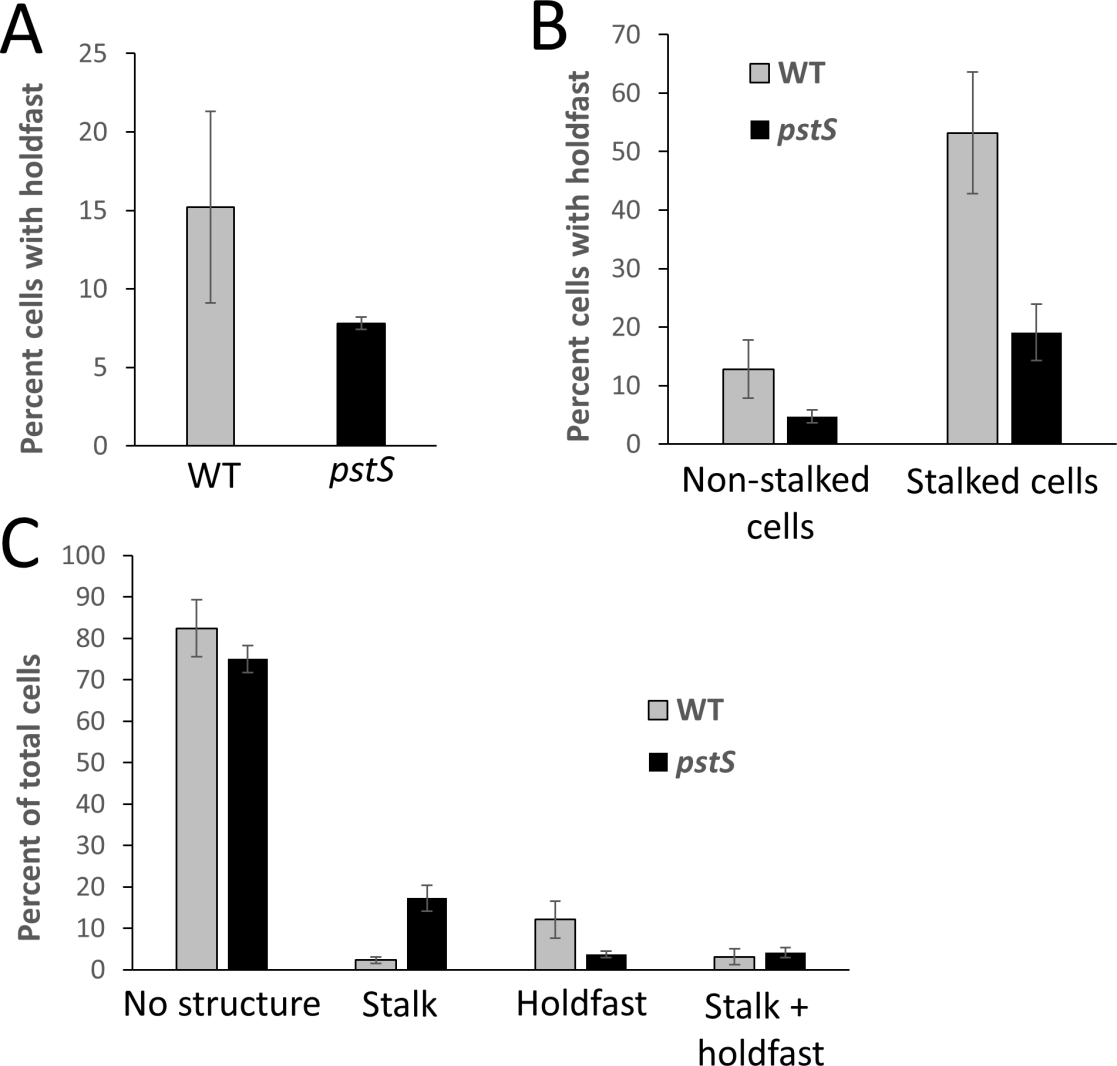
Incidence of stalk and/or holdfast detection in wild-type and Δ*pstS B*. *subvibrioides*. Wild-type and Δ*pstS* strains of *B*. *subvibrioides* were cultured in triplicate and labeled with fluorescently-conjugated Wheat Germ Agglutinin lectin, followed by phase contrast and epifluorescence microscopy. Cells were scored on whether they had a detectable stalk, holdfast, both, or neither. A) Percentage of the total population with a detectable holdfast. B) Percentage of non-stalked or stalked cells with a detectable holdfast. C) Percentage of total population that had no visible polar structure (i.e. no stalk or holdfast), stalk, holdfast, or both. Deletion of *pstS* led to an increase in stalk incidence, but the majority of stalked cells had no detectable holdfast.

In *C*. *crescentus*, stalks are often tipped with the adhesive polysaccharide holdfast. In fact, polar polysaccharide production is seen in other alphaproteobacteria as well [8, 17, 18]. Previous analysis of *B*. *subvibrioides* showed that this organism does produce a unipolar polysaccharide that stains with the same lectin as *C*. *crescentus* holdfast, strongly suggesting that *B*. *subvibrioides* also produces a holdfast [10]. However, holdfast incidence and its relation to stalk formation had not been analyzed. Fluorescently-conjugated lectin staining was performed on the *B*. *subvibrioides* wild-type and Δ*pstS* strains, and cells were scored as to whether they produced a visible stalk, detectable holdfast material, both, or neither (Fig 5).

**Fig 6:**
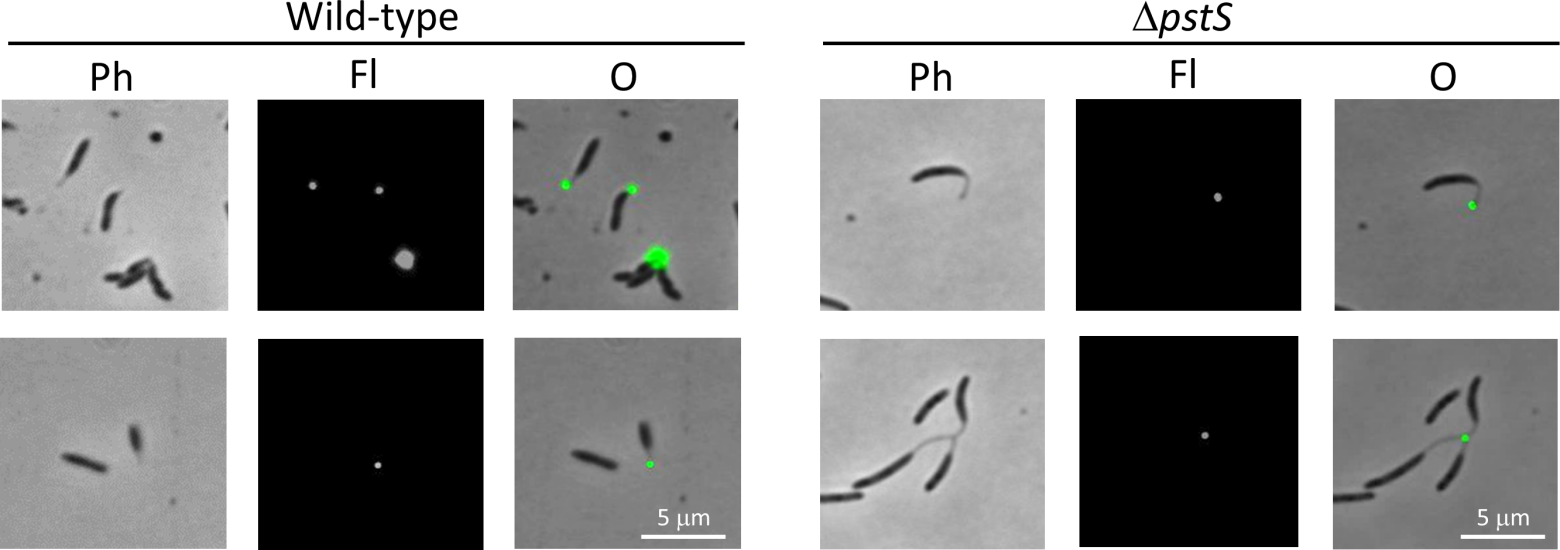
Holdfast was detected at the tip of stalks in wild-type and Δ*pstS B*. *subvibrioides* stalked cells. Staining of polysaccharide by fluorescently-conjugated wheat germ agglutinin lectin shows that holdfast material is located at the tip of the stalk when stalks are formed by *B*. *subvibrioides* wild-type or Δ*pstS* cells. Stained cells were imaged by phase contrast (Ph) and epifluorescence (Fl) microscopy, and images were overlaid (O). In some instances, several cells were joined at the holdfast forming a “rosette”, a common occurrence in *C*. *crescentus*.

As a percentage of the total population, *pstS* deletion caused a slight reduction in the number of cells producing a detectable holdfast (15.2 +/- 6.1% to 7.8 +/- 0.4%, Fig 5A); it is unclear if this is the result of altered Pho regulon signaling or due to the decrease in general fitness of that strain. When cells were categorized into non-stalked or stalked cells, wild-type and Δ*pstS* non-stalked cells had relatively low incidence of cells with labeled holdfasts (12.8 +/- 5.0% for wild-type, 4.7 +/- 1.1% for Δ*pstS*, Fig 5B). Interestingly, stalked cells in the wild-type population had a very high incidence of holdfast detection at 53.2 +/- 10.4%. It should be noted that because visibly stalked cells occur at such a low rate in the wild-type population the number of cells in this category is much smaller than in other categories. Yet, 127 visibly stalked cells were counted in this assay and more than half of them had detectable holdfast. These results suggest a correlation between stalk production and holdfast synthesis in the wild-type *B*. *subvibrioides* population. The percentage of visibly stalked cells with a detectable holdfast was much lower in the Δ*pstS* strain (19.1 +/- 4.8%) compared to wild-type. However, as a percentage of the total population, the incidence of cells with both detectable stalks and holdfasts was roughly equivalent between the two strains (3.1 +/- 1.9% for wild-type, 4.1 +/- 1.2% for Δ*pstS*, Fig 5C). Therefore, it appears that the *pstS* deletion leads to an increase in visible stalk incidence but the majority of those stalked cells do not have a detectable holdfast. Again, it is not clear if there is a direct causal link with Pho regulation. In all instances, in either wild-type or Δ*pstS* strains, where a stalk and holdfast were detected on the same cell the holdfast appears to be localized to the tip of the stalk (Fig 6); i.e. there were no observed cases of a holdfast at the base of a stalk.

### *B*. *subvibrioides* stalk biogenesis under nutrient-rich conditions appears not phase-variable, but may be reversible

The fact that a very small proportion of the total population of *B*. *subvibrioides* cells produce visible stalks under nutrient-rich conditions raises the intriguing possibility that stalk formation in this species could be phase variation regulated. Phase variation is a regulation pattern where a lineage of cells stably expresses a trait but occasionally and stochastically changes the expression of that trait. In a population of cells this results in a portion of the population expressing the trait and the rest of the population not expressing the trait. For example, *opvAB* expression in *Salmonella enterica* serovar Typhimurium is phase-regulated where in a population of cells some express *opvAB* which leads to smaller O-antigens; this increases resistance to bacteriophage that use O-antigens as a mechanism of infection, but also had reduced infection in a mouse model (reviewed in [19]). Cells not expressing *opvAB* had longer O-antigens and more pathogenicity, but also increased phage susceptibility. It is not uncommon for a phase-variable trait to be seen expressed at a low level in an otherwise homogenous population.

**Fig 7:**
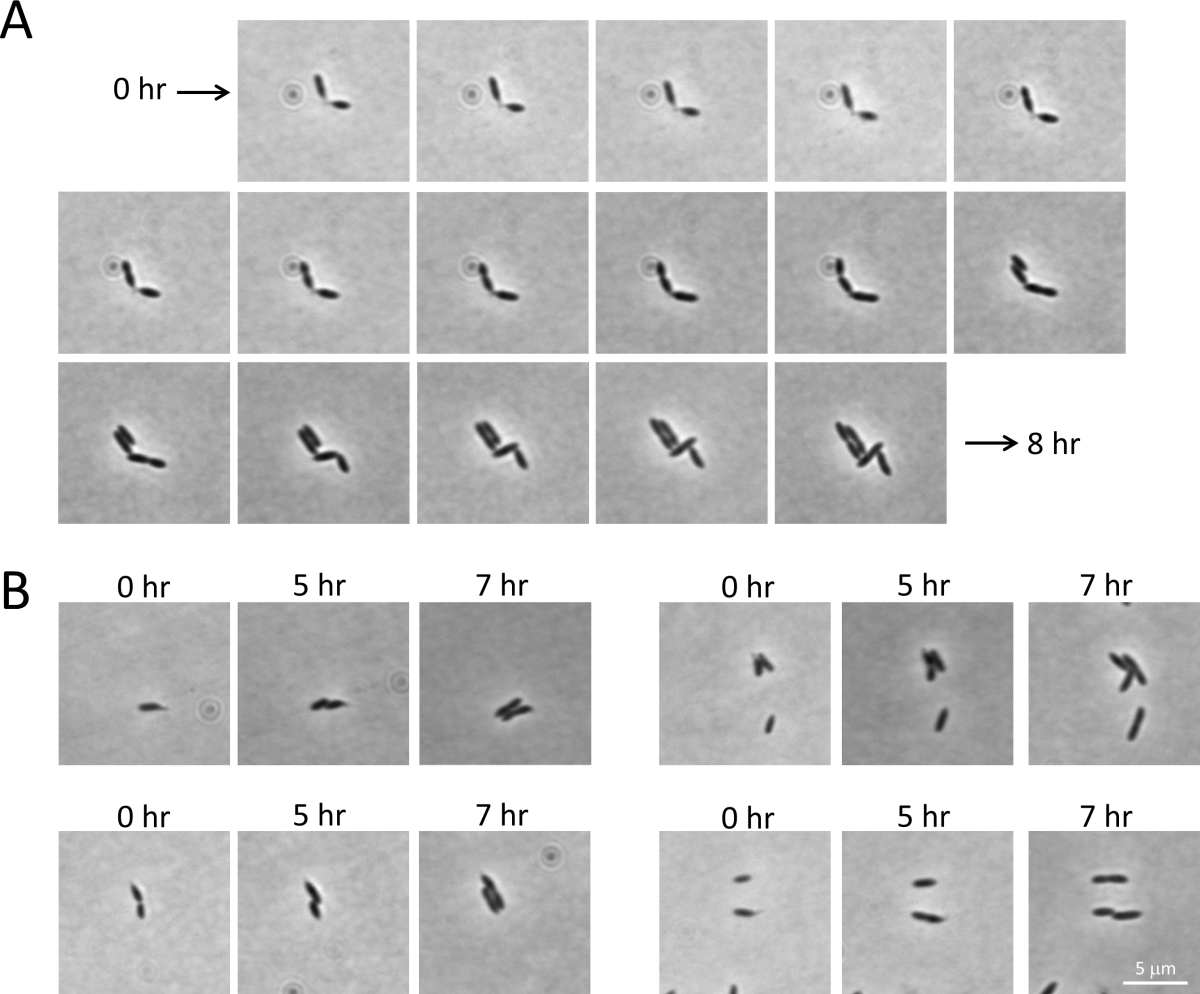
Time-lapse microscopy of stalked wild-type *B*. *subvibrioides* cells under nutrient rich conditions. Wild-type *B*. *subvibrioides* cultures were grown to mid-exponential phase in rich growth medium, and then mounted on rich medium agarose pads. The growth of 20 stalked cells was observed every 30 minutes for 8 hrs, typically covering two cell divisions. A) A representative time-lapse is presented, demonstrating two stalked cells growing and dividing, both producing non-stalked cells as well as seemingly losing their stalks. B) Selected images from time-lapse experiments showing *B*. *subvibrioides* cell potentially losing stalks.

To determine if stalk biogenesis under nutrient-rich conditions is phase regulated in *B*. *subvibrioides*, time lapse microscopy was carried out on stalked cells of *B*. *subvibrioides* in 2X PYE media. Phase variable traits are stably expressed in individual lineages with stochastic switching. Therefore, if stalk biogenesis is phase regulated, stalked cells should give rise to predominantly more stalked cells than non-stalked cells. The fate of 20 stalked cells was tracked for 8 hours, which typically covered two cell division events. A representative time lapse is presented in Fig 7A. Of the 20 stalked cells tracked, none produced visibly stalked cell progeny, suggesting that either stalk biogenesis is phase regulated with a very high switching rate, or, more likely, that stalk biogenesis is not phase regulated.

Not only did stalked cells produce non-stalked progeny, over the course of the time lapse 15 of 20 observed stalked cells appeared to lose their stalks. As shown in Fig 7A, two cells begin attached stalk-to-stalk, but by the end of the time lapse the poles of the cells are touching and no stalks are seen. Fig 7B shows other stalked cells that appear to lose their stalks over time. These results suggest the possibility that stalks can be remodeled in this organism. However, as cells grow on the agarose pad the cell bodies move relative to the surface, so it is possible that the small stalks of wild-type *B*. *subvibrioides* in rich media could get pushed under or otherwise hid by the expanding cell body. Without the ability to induce longer stalk formation in a controllable manner or specifically label stalk material, the ability of *B*. *subvibrioides* to remodel or disassemble the stalk is still undetermined.

**Fig 8:**
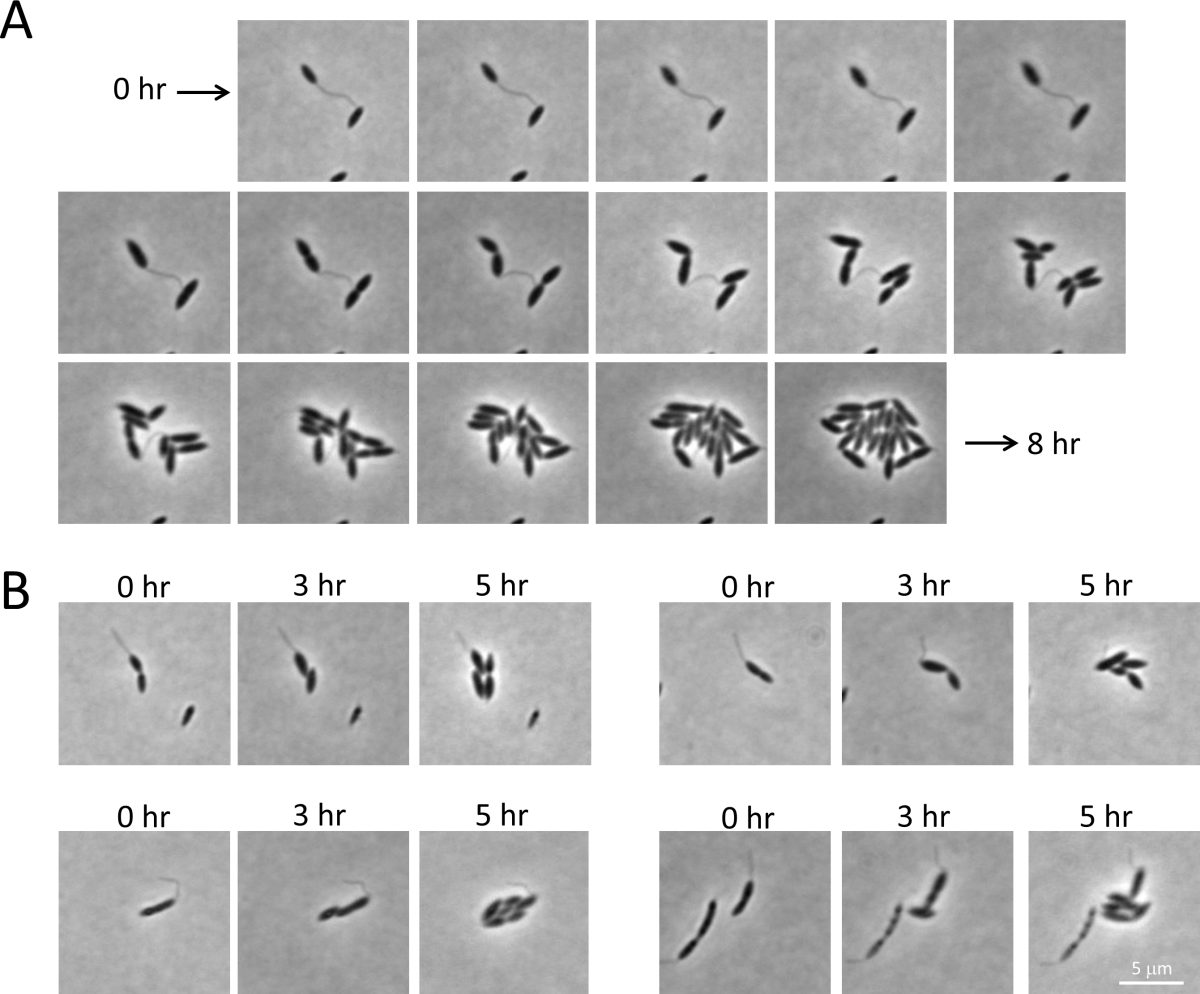
Time-lapse microscopy of *C*. *crescentus* cells shifted from nutrient-poor to nutrient-rich conditions. Wild-type *C*. *crescentus* was cultured in phosphate-limited HIGG growth media to mid-exponential phase to induce longer than usual stalks, then cells were mounted on rich medium agarose pads. The growth of 20 stalked cells was observed every 30 minutes for 8 hrs. A) A representative time-lapse is presented where a cell with a very long stalk progresses through multiple divisions while appearing to maintain stalk length. B) Selected images from time-lapse experiments showing cells with long stalks maintaining stalk length.

To assess the possibility of stalk remodeling in *C*. *crescentus*, wild-type cells were grown in HIGG minimal media containing limiting phosphate levels (30 μM). Cells grown under these conditions generate longer than usual stalks. Cells from these cultures were then placed on 2X PYE agarose pads and imaged every 30 minutes for 8 hours. As wild-type *C*. *crescentus* often produces stalks under nutrient-rich conditions, it would not be expected that a nutrient shift would result in complete stalk disassembly. However, the long stalks formed under phosphate starvation may be shortened in response to nutrient up-shift. A representative time course as well as other select images are shown in Fig 8. It does not appear that *C*. *crescentus* cells shorten their stalks once placed in nutrient-rich conditions. The same result was obtained following *C*. *crescentus* cell cultured in PYE instead of HIGG (S1 Fig). Therefore, if *B*. *subvibrioides* does remodel its stalk, it does not appear to be a behavior conserved in *C*. *crescentus*.

### Many *C*. *crescentus* stalk-associated proteins are not conserved in related organisms

As an extension of the cell wall, stalk synthesis is an exercise in localized peptidoglycan synthesis. In *C*. *crescentus*, two bactofilin proteins BacA and BacB form a polymeric sheet associated with the membrane close to the stalked pole [20]. This polymer mediates the recruitment of a Class A bifunctional penicillin binding protein PbpC. PbpC is one of five bifunctional PBP's in *C*. *crescentus* and has the largest responsibility in stalk synthesis; disruption of *pbpC* leads to a 25% reduction in stalk length, though clearly other *C*. *crescentus* PBPs can substitute for PbpC's stalk synthesis role [20, 21]. The composition of the stalk compartment is highly regulated; the core and inner membrane of the stalk are largely devoid of proteins found in the cell body cytoplasm and inner membrane, as determined by proteomic analysis as well as GFP-labeling of specific proteins [11, 22, 23]. While the stalk periplasm and outer membrane have similar protein compositions to the cell body periplasm and outer membrane, the stalk is compartmentalized by protein diffusion barriers called crossbands which prevent macromolecular exchange between the periplasm/outer membrane areas of stalk and cell body [18]. Some proteins are specifically localized to the stalk, such as the inner membrane protein StpX. The exact role of StpX in the cell is not known, but it is highly abundant in, and specifically localized to, the stalk. While other *C*. *crescentus* PBPs can substitute for PbpC in stalk synthesis, they cannot substitute for PbpC's role in embedding StpX into the stalk compartment [24]. In wild-type *C*. *crescentus*, StpX is localized almost exclusively to the stalk (a process that depends on active stalk synthesis), while in a *pbpC* or *bacA* mutant StpX is found in the inner membrane of both the stalk and cell body.

On a morphological level, the *B*. *subvibrioides* stalk resembles the *C*. *crescentus* stalk. The *B*. *subvibrioides* stalk appears contiguous with cell wall, a holdfast is produced at the tip, and crossbands have even been observed (S2 and S3 Figs); though it should be noted crossbands have only been observed in a Δ*gcrA* mutant and the ability of wild-type *B*. *subvibrioides* to produce crossbands has yet to be investigated. However, genomic analysis reveals surprisingly little conservation of known *C*. *crescentus* stalk-associated proteins in related organisms. A conservation analysis of genes encoding several proteins listed above in seven *Brevundimonas* species with sequenced genomes, as well as two *Asticcacaulis* species (another genus in the *Caulobacter* clade with stalked organisms) is presented in Table 1. Homologs were identified by BlastP and required Bi-directional Best Hit to be considered an ortholog. All identified homologs were orthologs and had significant similarity across the full length of the *C*. *crescentus* protein sequences; any other potential homolog only shared similarity with portions of the *C*. *crescentus* sequences. Given that all the species are quite closely related, the ease in finding these orthologs was expected.

**Table 1:** Conservation of stalk-related genes in members of the Caulobacterales clade^1^.

*C*. *crescentus*	BacA	BacB	StpA	StpB	StpC	StpD	StpX
*B*. *subvibrioides*	Bresu_2189	N	Bresu_2758	N	N	N	N
*B*. *abyssalis*	MBE-BAB_1295	N	MBE-BAB_1178	N	N	N	N
*B*. *aveniformis*	G391DRAFT_1601	N	N	N	N	N	N
*B*. *bacteroides*	Q333DRAFT_1899	N	Q333DRAFT_2084	N	N	N	N
*B*. *diminuta*	BDIM_12520	N	N	N	N	N	N
*B*. *nasdae*	Ga0063995_12410	N	N	N	N	N	N
*B*. *naejangsanensis*	DA69DRAFT_01149	N	N	N	N	N	N
*A*. *biprosthecum*	ABI_34180	N	ABI_10880	ABI_10870	N	N	N
*A*. *excentricus*	Astex_2752	N	Astex_0986	Astex_0987	N	N	N

^1^ locus tags of orthologs are given if present, N indicates no homolog is detectible.

All species listed had a homolog for the bactofilin BacA, but none for BacB. However, in *C*. *crescentus* PbpC localization is dependent on BacA but not BacB [20, 24], suggesting BacA is more functionally important. In *C*. *crescentus* four proteins make up the crossband diffusion barriers: StpA, StpB, StpC, and StpD, with StpA being necessary for the assembly of all other crossband proteins [25]. The *Asticcacaulis* species have homologs of only StpA and StpB. StpB, StpC and StpD are not found in any *Brevundimonas* species, and only three of the seven *Brevundimonas* species (including *B*. *subvibrioides*) have homologs for StpA. These results suggest that either *B*. *subvibrioides* crossbands are purely composed of StpA, or crossbands in that organism have other components not conserved with *C*. *crescentus*. With the lack of conserved components, it is also possible that crossbands in *B*. *subvibrioides* are not as functional at compartmentalization as *C*. *crescentus*. StpX is not found in any species outside the *Caulobacter* genus. Lastly, it is also worth noting that *B*. *subvibrioides* has two Class A PBP homologs, neither of which is orthologous to PbpC. Bresu_0560 is orthologous to PbpX (CC_0252), the PBP that has the largest role in cell growth in *C*. *crescentus* but also is involved in stalk biogenesis; Bresu_2191 is orthologous to PbpY (CC_1875), a PBP that has been implicated in cell growth, division and stalk biogenesis [21]. These results suggest there may be significant differences in the biogenesis and maintenance of stalks between *C*. *crescentus* and other stalked-synthesizing organisms even though they are closely related.

Regulation of the developmental stalk synthesis program has been tied to the sigma-54 activationg ShkA-ShpA-TacA phosphorelay as well as a regulatory target of the relay StaR; mutants of the phosphorelay do not produce stalks under high phosphate conditions, and *staR* mutants have short stalks while *staR* overexpression leads to long stalks [5, 26, 27]. Analysis of the *Brevundimonas* and *Asticcacaulis* genomes revealed orthologs for ShkA, ShpA, TacA and StaR in all genomes with the exception *B*. *diminuta* where orthologs could not be found for ShkA or StaR. There is a potential homolog for StaR in *B*. *diminuta* (BDIM_27560) with a BLASP value of e^-26^ and 76% amino acid identity, but the homology was largely confined to the N-terminal portion of the predicted protein. As the exact relationship between the phosphorelay and stalk biogenesis is not understood, the significance of phosphorelay conservation is unclear.

### *B*. *subvibrioides* PstA-GFP is detectable in the stalk

**Fig 9:**
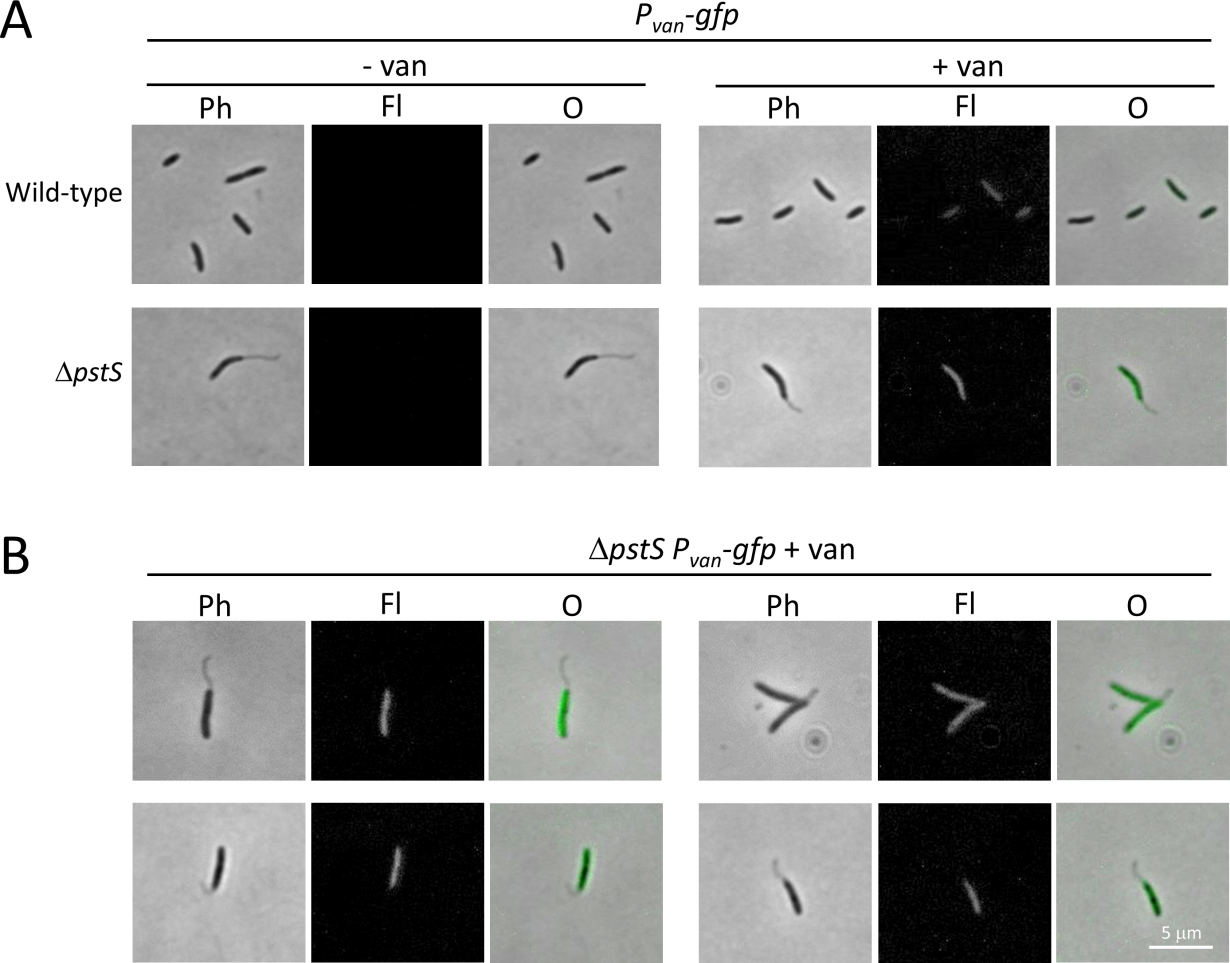
Soluble GFP is not detected in *B*. *subvibrioides* stalks. The plasmid pRVGFPC-2 was moved into wild-type and Δ*pstS* strains; upon vanillate induction soluble GFP is produced. This construct was moved into wild-type and Δ*pstS B*. *subvibrioides* strains. A) Cells were imaged in the absence (- van) or presence (+ van) of vanillate. Imaging was performed by phase contrast (Ph) and epifluorescence (Fl) microscopy, and the images were overlaid (O). Upon examination, cytoplasmic GFP does not enter the stalk compartment. B) Additional examples of Δ*pstS* cells with soluble GFP absent from stalks.

**Fig 10:**
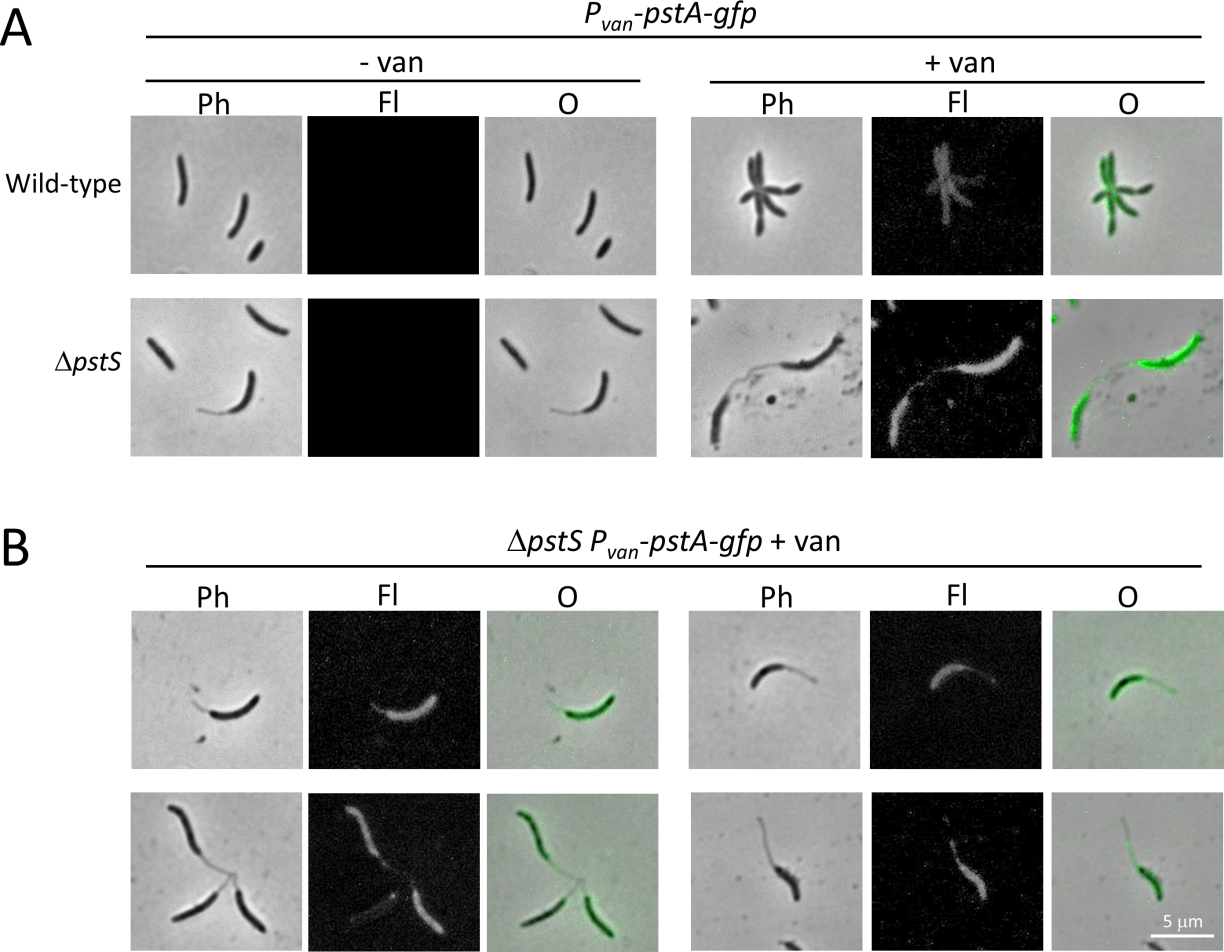
PstA-GFP is detectable in some *B*. *subvibrioides* stalks. The *B*. *subvibrioides pstA* gene was cloned into plasmid pRVGFPC-2, a replicating plasmid that creates a C-terminal GFP fusion as well as place the gene under a vanillate inducible promoter. This construct was moved into wild-type and Δ*pstS B*. *subvibrioides* strains. A) Cells were imaged in the absence (- van) or presence (+ van) of vanillate. Imaging was performed by phase contrast (Ph) and epifluorescence (Fl) microscopy, and the images were overlaid (O). PstA-GFP is detectable in some stalk compartments. B) More examples of PstA-GFP imaging. Top left and right: examples of fluorescence detected in stalks. Bottom left: examples of fluorescence not detected in stalks. Bottom right: example of fluorescence detected in a portion of a stalk.

Protein entry into the *C*. *crescentus* stalk is a regulated process. Proteomic analysis of *C*. *crescentus* stalks revealed that outer membrane and periplasmic stalk protein composition was very similar that of the same compartments of the cell body, but there were remarkably few detectable inner membrane or cytoplasmic proteins [22, 23]. Notably, only one protein component of the multi-protein phosphate transporter (PstB) was detectable in the stalk inner member, and GFP-tagged PstA (another phosphate transporter component) was found in the cell body but excluded from the stalk [23], which combined with other data has begun to cast doubt on the ability of the stalk to function in phosphate uptake (see Discussion). To begin examining the extent of *B*. *subvibrioides* stalk compartmentalization, plasmid constructs producing either soluble GFP or PstA-GFP when induced with vanillate were moved into wild-type and Δ*pstS* strains. Cultures were then grown in the absence or presence of vanillate and imaged (Figs 9 and 10). In the absence of vanillate no fluorescence could be visualized. When grown in the presence of vanillate many cells displayed at least some induction of fluorescence, though the level of induction was variable. Some cells were brightly fluorescent, some had no fluorescence, and others had intermediate levels of fluorescence, suggesting that vanillate induction is not functional in all cells. The vanillate degradation pathway found in *C*. *crescentus* (and *Asticcacaulis* species) appears to be lost in the *Brevundimonas* genus; therefore, the ability of vanillate to enter into cells may be variable. Induction still functions in *B*. *subvibrioides* because the *vanR* repressor is encoded on the plasmid itself [13].

In all cells displaying fluorescence, no subcellular localization was observed and fluorescence was evenly distributed in the cell body; this is expected given the proteins involved. In the case of soluble GFP, fluorescence was confined to the cell body and never observed in stalks (Fig 9). Statistical analysis revealed no significant difference between the fluorescence in stalks of cells expressing GFP and background fluorescence (p = 0.72), supporting the observation that GFP is unable to enter the stalked compartment of *B*. *subvibrioides*. However, as the cell body fluorescence was the lowest of the constructs tested, it cannot be definitively concluded that GFP is unable to enter the stalk. Yet even in the most fluorescent cells, where cell body fluorescence was comparable to other constructs that demonstrated stalk fluorescence (see below), no stalk fluorescence could be observed (Fig 9B). These results are consistent with a model where soluble proteins are excluded from the *B*. *subvibrioides* stalk, which itself is consistent with the *C*. *crescentus* model.

A different result was obtained with the PstA-GFP construct. Here, fluorescence was detectable in the stalked compartment (Fig 10). Detection was variable, with some cells displaying fluorescence along the full length of the stalk (Fig 10B, top), some with no stalked fluorescence (Fig 10B, bottom left) and some with fluorescence in only the portion of the stalk proximal to the cell body (Fig 10B, bottom right). Even with the variability of detection, quantification of stalk fluorescence for this construct was statistically significant from background (p = 3.92e-12), demonstrating that PstA-GFP is able to enter the stalk compartment in *B*. *subvibrioides*. While this result does not demonstrate that the rest of the phosphate transporter components can enter the stalk or that the stalk inner membrane is capable of phosphate transport, this result does stand in stark contrast to *C*. *crescentus* where PstA-GFP was excluded from the stalk compartment [23]. It is possible that the inner membrane of *B*. *subrivibrioides* stalks is not as regulated as *C*. *crescentus*, and may be functional at phosphate uptake (see Discussion).

### *C*. *crescentus* StpX is able to enter the stalk in *B*. *subvibrioides*

**Fig 11:**
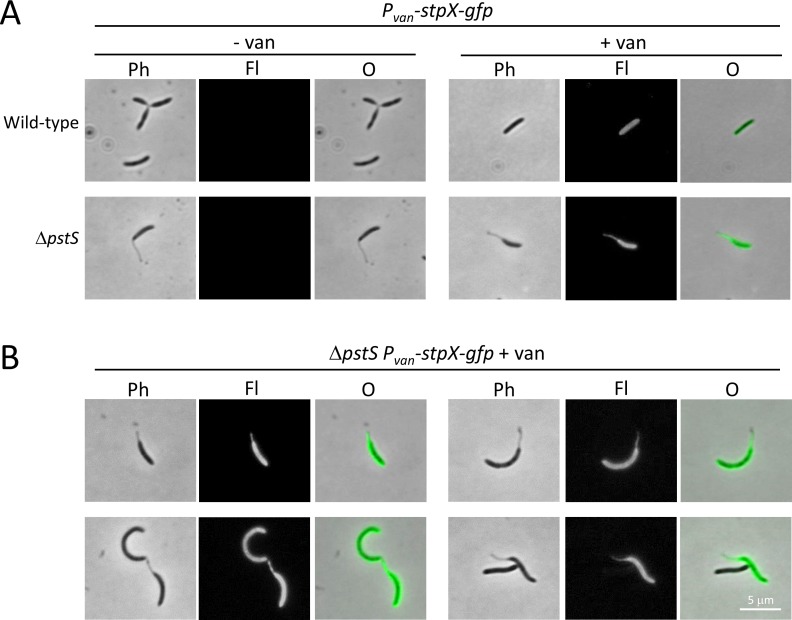
*B*. *subvibrioides* prevents soluble GFP from entering the stalk but allows StpX into the stalk. The *C*. *crescentus stpX* gene was cloned into plasmid pRVGFPC-2, a replicating plasmid that creates a C-terminal GFP fusion as well as place the gene under a vanillate inducible promoter. This construct was moved into wild-type and Δ*pstS B*. *subvibrioides* strains. A) Cells were imaged in the absence (- van) or presence (+ van) of vanillate. Imaging was performed by phase contrast (Ph) and epifluorescence (Fl) microscopy, and the images were overlaid (O). Upon examination, fluorescence was detected in both the stalk and cell body. B) Additional examples of fluorescence in stalks and cell body.

The *C*. *crescentus* StpX protein has an unusual property in that it is one of the few inner membrane proteins that can enter the stalk compartment in that organism [24, 28, 29], yet it does not appear to be conserved outside the *Caulobacter* genus. To determine the behavior of *C*. *crescentus* StpX when placed in *B*. *subvibrioides*, a plasmid construct with *stpX-gfp* placed under a vanillate-inducible promoter was moved into wild-type and Δ*pstS B*. *subvibrioides* strains (Fig 11).

StpX-GFP was found in both the cell body and the stalk and statistical analysis revealed that stalk fluorescence was significantly different from background fluorescence (p = 1.18e-46). Therefore, *C*. *crescentus* StpX is able to enter the *B*. *subvibrioides* stalk compartment. One potential explanation for these results is that whatever mechanism segregates inner membrane proteins and allows StpX to enter the stalk compartment in *C*. *crescentus* is also conserved in *B*. *subvibrioides* despite the fact that many stalk-associated proteins, including StpX itself, are not conserved in *B*. *subvibrioides*. Alternatively, given the PstA-GFP results, it is possible that *B*. *subvibrioides* does not regulate inner membrane protein exchange between cell body and stalk compartments. Either option is potentially interesting.

StpX-GFP fluorescence in both *B*. *subvibrioides* cell body and stalk is reminiscent of a particular *C*. *crescentus* mutant phenotype. In wild-type *C*. *crescentus*, StpX is localized predominantly to the stalk with little cell body detection, but in a *pbpC* mutant StpX-GFP was found extensively in both the stalk and cell body. As *B*. *subvibrioides* does not have a PbpC ortholog, perhaps it is not surprising to find *C*. *crescentus* StpX in both *B*. *subvibrioides* compartments. Yet, this result is noteworthy in that in the *C*. *crescentus pbpC* mutant photobleached stalk StpX-GFP was rapidly replenished by cell body StpX-GFP, demonstrating a case where an inner membrane protein could freely diffuse between the cell body and stalk compartments [24]. It was not determined if other inner membrane proteins could also enter the stalk compartment in that strain.

## Discussion

The exact function of the stalk is unknown. Because *C*. *crescentus* typically lives in phosphate-limiting environments and the stalk is lengthened in response to phosphate starvation, it was historically thought that the stalk was simply a phosphate antenna; a way of increasing the surface area of the cell without significantly increasing the volume. However, recent advances in understanding stalk synthesis and composition has forced a reassessment of this theory. Phosphate diffuses through outer membrane porins in *C*. *crescentus* and is then captured by the periplasmic protein PstS, which brings it to the PstCAB transporter. The first indication that the stalk might not function directly in phosphate uptake was the finding that Pst transporter proteins could not be found in the inner membrane of the stalk [23]. It was therefore thought that PstS captures phosphate in the stalk periplasm but the complex then diffuses through the stalk periplasm into the cell body periplasm where it then interacts with the Pst transporter complex in the inner membrane. However, with the discovery that crossbands are protein diffusion barriers and effectively compartmentalize the stalk, it prevents stalk PstS from interacting with the Pst transporter [25]. Phosphate bound to PstS in the stalk would in fact likely disassociate and leave the cell before reaching the Pst transporter [30]. Therefore, it does not appear likely that the stalk functions directly in phosphate uptake. In regards to other nutrients, the *C*. *crescentus* stalk outer membrane has a larger number of transporters, but the stalk inner membrane is virtually devoid of transporters [22, 23], so other nutrients transported into the stalk periplasm face much the same problem as phosphate. It is not clear if these nutrients could diffuse through the stalk periplasm to reach the cell body inner membrane and be transported into the cytoplasm before they diffuse out of the cell. Yet, even if they do not in *C*. *crescentus*, the PstA-GFP results from this study suggest that in other organisms the stalk may be more directly involved in nutrient uptake.

Stalk biogenesis is clearly induced by phosphate starvation, so if it does not function directly in phosphate uptake in *C*. *crescentus*, it begs the question why. With the incredibly strong adhesive strength of the polar holdfast, *C*. *crescentus* and related organisms are adept at surface attachment, but any cells that become nutrient limited lack the ability to move to a new environment because they are strongly attached. There are at least two ways in which *C*. *crescentus* cells could become nutrient limited [30]. Cells inside a biofilm can become limited just by sheer numbers of cells and competition for resources. Additionally, cells attached to an abiotic surface can easily become nutrient limited. Close to a solid-liquid interface is a “boundary layer” of water. This water is static and molecular movement through this layer is purely based on diffusion. Therefore, cells attached to abiotic surfaces can become diffusion limited as they deplete all local resources. In either case, a useful solution to nutrient depletion would be to extend the cell body away from the attachment point, thereby putting the cell body into more dynamic fluid flow, which would presumably increase exposure to nutrients. The production of a stalk at one pole would serve this purpose. If this hypothesis is correct, then lengthening the stalk in response to phosphate starvation is not a mechanism to increase phosphate uptake capability, but instead phosphate starvation is used as a proxy for nutrient availability and stalk lengthening adjusts the proximity of the cell to its surface attachment point.

Based on these points, a phosphate starvation program of stalk synthesis is well justified. What is not clearly justified is a developmental program for stalk synthesis. The research presented here suggests a model where *B*. *subvibrioides* synthesizes a stalk predominantly on an as-need basis, and potentially disassembles the stalk when no longer needed. This research also suggests that this model may be prevalent among the *Caulobacter* and *Brevundimonas* genera, and that the developmental stalk program of *C*. *crescentus* may in fact be an outlier. If so, why have a developmental stalk biogenesis program? Is there an advantage to producing a stalk even when the cell is not attached to a surface or starving for nutrients?

It is not clear how much the mechanics of stalk formation are conserved between *C*. *crescentus* and *B*. *subvibrioides*. There is remarkably little conservation of known stalk-associated proteins. If there are important functional differences between the stalks of the two organisms, perhaps the differences are driven by the presence/absence of a developmental stalk program; i.e. the formation or function of the stalk may be different when it is formed under multiple nutrient conditions like in *C*. *crescentus*, instead of forming principally under phosphate starvation conditions as suggested by this research in *B*. *subvibrioides*. Clearly greater examination of the *B*. *subvibrioides* stalk is necessary.

## Supporting information

S1 Table Strains. (DOCX)

S1 Fig Timelapse of wild-type *C*. *crescentus* under nutrient-rich conditions. *C*. *crescentus* was cultured in nutrient-rich PYE media prior to spotting on 2XPYE agarose pads, and was visualized every 30 minutes for 8 hours.
(TIF)

S2 Fig Transmission electron micrographs of *B*. *subvibrioides* cells displaying crossbands. TEM images was taken of a *B*. *subvibrioides* Δ*gcrA* cells that show stalks with crossbands.
(TIF)

S3 Fig Transmission electron micrographs of *B*. *subvibrioides* cells displaying crossbands. TEM images was taken of a *B*. *subvibrioides* Δ*gcrA* cells that show stalks with crossbands.
(TIF)
