# Supplementary material for: Stalk formation of Brevundimonas and how it compares to Caulobacter crescentus
Source: PLoS One. 2017 Sep 8;12(9):e0184063. doi: 10.1371/journal.pone.0184063 (PMC5590869; doi:10.1371/journal.pone.0184063)
Supplement: S1 Table — (DOCX) [file pone.0184063.s001.docx]

**Table S-1: Strains**

| Organism | Genotype | Source |
| --- | --- | --- |
| *Escherichia coli* | DH5α | Thermo-Fisher |
| *Caulobacter crescentus* | NA1000 | [[1](#_ENREF_1)] |
|  | NA1000 *pstS*::miniTn5 | [[2](#_ENREF_2)] |
| *Brevundimonas subvibrioides* | Wild-type | ATCC15264 |
|  | Δ*pstS* | This study |
|  | Δ*phoB* pRVMCS-5 P_van_-*phoB* | This study |
|  | pRVGFPC-2 | This study |
|  | pRVGFPC-2 P_van_-*pstA-gfp* | This study |
|  | pRVGFPC-2 P_van_-*stpX-gfp* | This study |
|  | Δ*pstS* pRVGFPC-2 | This study |
|  | Δ*pstS* pRVGFPC-2 P_van_-*pstA-gfp* | This study |
|  | Δ*pstS* pRVGFPC-2 P_van_-*stpX-gfp* | This study |
| *Caulobacter henricii* | Wild-type | ATCC15253 |
| *Caulobacter sp. K31* | Wild-type | C. Stephens |
| *Caulobacter fusiformis* | Wild-type | ATCC15257 |
| *Caulobacter segnis* | Wild-type | ATCC21756 |
| *Brevundimonas abyssalis* | Wild-type | CECT8073 |
| *Brevundimonas alba* | Wild-type | ATCC15265 |
| *Brevundimonas aveniformis* | Wild-type | DSM17977 |
| *Brevundimonas bacteroides* | Wild-type | ATCC19090 |
| *Brevundimonas basaltis* | Wild-type | DSM25335 |
| *Brevundimonas bullata* | Wild-type | DSM7126 |
| *Brevundimonas diminuta* | Wild-type | ATCC11568 |
| *Brevundimonas halotolerans* | Wild-type | DSM8802 |
| *Brevundimonas intermedia* | Wild-type | DSM4732 |
| *Brevundimonas kwangchunensis* | Wild-type | DSM17033 |
| *Brevundimonas lenta* | Wild-type | DSM23960 |
| *Brevundimonas mediterranea* | Wild-type | DSM14878 |
| *Brevundimonas naejengsanensis* | Wild-type | DSM23858 |
| *Brevundimonas nasdae* | Wild-type | DSM14572 |
| *Brevundimonas poindexterae* | Wild-type | DSM24277 |
| *Brevundimonas terrae* | Wild-type | DSM17329 |
| *Brevundimonas vesicularis* | Wild-type | DSM7226 |

**References**

1. Evinger M, Agabian N. Envelope-associated nucleoid from *Caulobacter crescentus* stalked and swarmer cells. J Bacteriol. 1977;132(1):294-301. Epub 1977/10/01. PubMed PMID: 334726.

2. Gonin M, Quardokus EM, O'Donnol D, Maddock J, Brun YV. Regulation of stalk elongation by phosphate in *Caulobacter crescentus*. J Bacteriol. 2000;182(2):337-47. Epub 2000/01/12. PubMed PMID: 10629178.
