# Supplementary figures and images for: Stalk formation of Brevundimonas and how it compares to Caulobacter crescentus
Source: PLoS One. 2017 Sep 8;12(9):e0184063. doi: 10.1371/journal.pone.0184063 (PMC5590869; doi:10.1371/journal.pone.0184063)

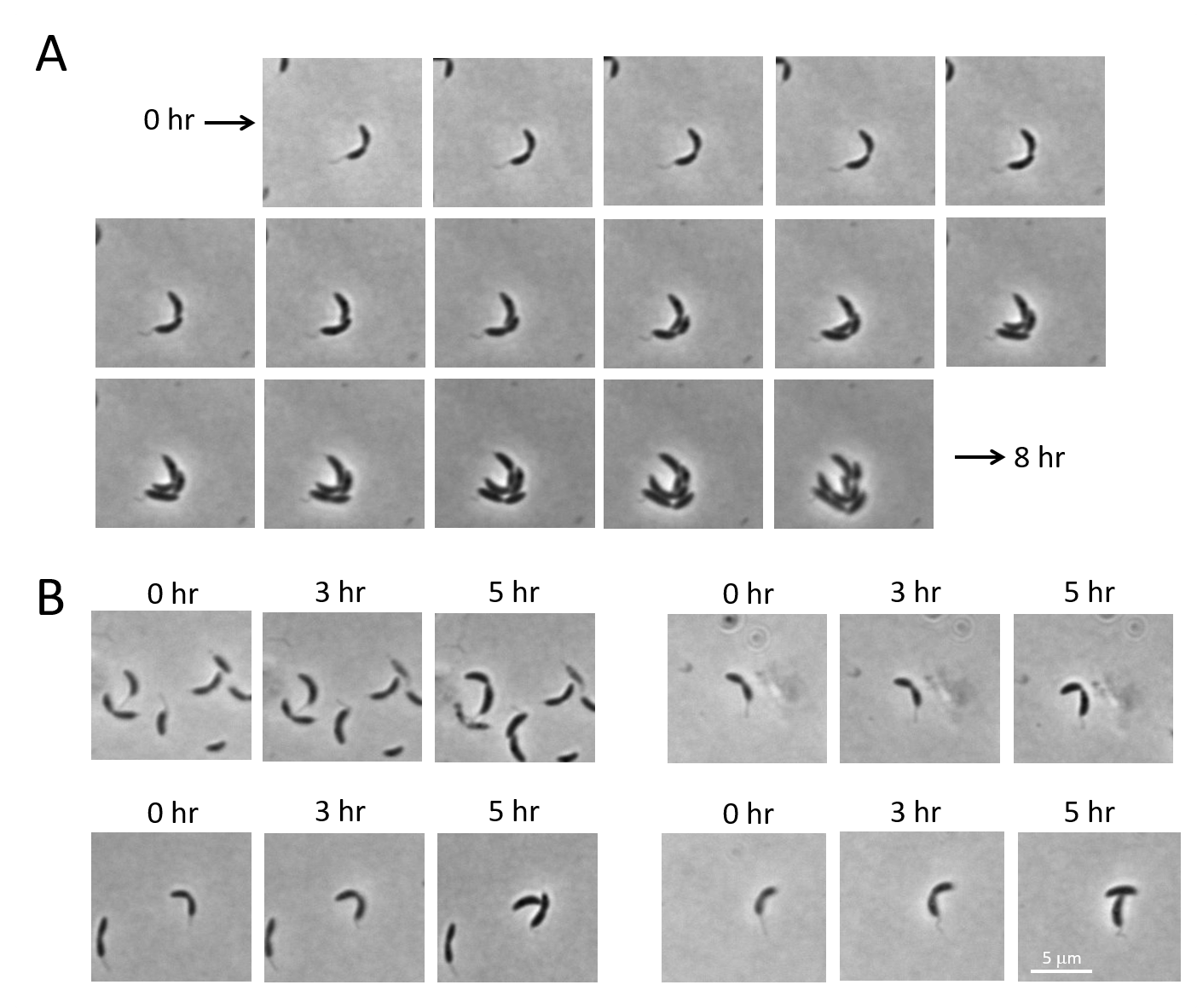

Supplement: S1 Fig — C. crescentus was cultured in nutrient-rich PYE media prior to spotting on 2XPYE agarose pads, and was visualized every 30 minutes for 8 hours. (TIF) [file pone.0184063.s002.tif]
